# Semliki Forest virus strongly reduces mosquito host defence signaling

**DOI:** 10.1111/j.1365-2583.2008.00834.x

**Published:** 2008-09-22

**Authors:** R Fragkoudis, Y Chi, R W C Siu, G Barry, G Attarzadeh-Yazdi, A Merits, A A Nash, J K Fazakerley, A Kohl

**Affiliations:** * The Roslin Institute & Royal (Dick) School of Veterinary Studies, College of Medicine & Veterinary Medicine, The University of EdinburghSummerhall, Edinburgh EH9 1QH, Scotland, UK; † Institute of Technology, University of TartuNooruse Str. 1, Tartu 50411, Estonia

**Keywords:** Semliki Forest virus, mosquito cells, host response, cell signaling, virus/host interactions

## Abstract

The *Alphavirus* genus within the *Togaviridae* family contains several important mosquito-borne arboviruses. Other than the antiviral activity of RNAi, relatively little is known about alphavirus interactions with insect cell defences. Here we show that Semliki Forest virus (SFV) infection of *Aedes albopictus-*derived U4.4 mosquito cells reduces cellular gene expression. Activation prior to SFV infection of pathways involving STAT/IMD, but not Toll signaling reduced subsequent virus gene expression and RNA levels. These pathways are therefore not only able to mediate protective responses against bacteria but also arboviruses. However, SFV infection of mosquito cells did not result in activation of any of these pathways and suppressed their subsequent activation by other stimuli.

## Introduction

The *Alphavirus* genus within the *Togaviridae* family contains several important mosquito-borne arboviruses including Chikungunya (Charrel *et al*., 2007) and the equine encephalitis viruses (Weaver & Barrett, 2004). Replication of the prototype Sindbis and Semliki Forest viruses is well understood (Strauss & Strauss, 1994; Kaariainen & Ahola, 2002). The genome consists of a positive-stranded RNA with a 5′ cap and 3′ polyA tail. The 5′ two-thirds encodes the nonstructural polyprotein P1234, which is translated from the viral genome after virus entry and cleaved into four replicase proteins nsP1–4 (Merits *et al*., 2001; Kim *et al*., 2004; Lulla *et al*., 2006). The structural polyprotein is encoded in the 3′ third of the genome, and is cleaved into capsid and glycoproteins after translation from a subgenomic mRNA. Cytoplasmic replication complexes are associated with cellular membranes (Salonen *et al*., 2005).

Viruses and other intracellular pathogens have to overcome cellular host defences to replicate efficiently. In vertebrates, detection of viral signatures, for example dsRNA, is an important trigger of antiviral defences (Haller *et al*., 2006; Randall & Goodbourn, 2008).

Little is known about how invertebrate cells react to arbovirus infections. Establishment of persistent infections in invertebrate cells, as opposed to cell death in vertebrate cells, suggests fundamental differences (Brown, 1984; Higgs, 2004). dsRNA is detectable in alphavirus-infected mosquito cells (Stollar *et al*., 1972). Unlike in shrimp (Robalino *et al*., 2007) and *Lepidoptera* (Hirai *et al*., 2004), there is however no indication that control dsRNAs used in RNAi experiments stimulate antiviral responses in mosquitoes (Keene *et al*., 2004; Franz *et al*., 2006), or transfected mosquito cells (Caplen *et al*., 2002). To date, RNAi is the best-studied cellular mechanism known to prevent or limit arbovirus replication in mosquitoes and mosquito cells (Keene *et al*., 2004; Campbell *et al*., 2008).

Other invertebrate innate host defences have been most extensively studied in *Drosophila melanogaster*; pathogen recognition receptors and signaling pathways have been described (Silverman & Maniatis, 2001; Ferrandon *et al*., 2004; Royet, 2004). Fungi and Gram-positive bacteria activate a Toll-dependent pathway, Gram-negative bacteria the IMD and JNK (branching off IMD) pathways, resulting in production of antibacterial molecules such as defensins (Silverman *et al*., 2003). Mechanisms of insect innate immune signaling (Eggleston *et al*., 2000; Shin *et al*., 2003; Christophides *et al*., 2004; Osta *et al*., 2004; Bian *et al*., 2005; Shin *et al*., 2005; Meredith *et al*., 2006; Shin *et al*., 2006; Waterhouse *et al*., 2007; Warr *et al*., 2008) as well as RNAi (Sanchez-Vargas *et al*., 2004) seem to be conserved in *Anopheles* and *Aedes* mosquitoes, though there are differences at the molecular level between *Anopheles* and *Drosophila*, possibly due to different immune challenges (Christophides *et al*., 2002; Aguilar *et al*., 2005).

Experiments with pathogenic *Drosophila* viruses have shown that the *Drosophila* Toll (Zambon *et al*., 2005) and STAT (Dostert *et al*., 2005) pathways are involved in responses to infection. In mosquito cells, STAT proteins (Christophides *et al*., 2002; Lin *et al*., 2004) are also activated after bacterial challenge (Barillas-Mury *et al*., 1999).

Recently, a heat shock protein was shown to downregulate O’nyong-nyong alphavirus replication in *Anopheles gambiae* cells (Sim *et al*., 2007), while the Toll pathway controls Dengue virus infection (Xi *et al*., 2008). Genomic studies have used gene arrays to analyse the transcriptome of mosquito and thrips larvae (Medeiros *et al*., 2004; Sanders *et al*., 2005; Sim *et al*., 2005). In mosquito midguts infected with Sindbis virus (Sanders *et al*., 2005), early Toll and (possibly) IMD pathway component upregulation, late JNK activation and RNAi (*dicer-2*) downregulation were observed. Thrips larvae infected with the plant-infecting tomato spotted wilt bunyavirus show upregulation of Toll, IMD and JNK pathways (Medeiros *et al*., 2004). These effects might not directly be related to virus infection, as complex secondary responses such as cytokines or other ‘danger’ signals may also be relevant in tissues.

As the interplay between mosquito cell signaling pathways and alphaviruses is not understood, we investigated the effect of SFV infection on mosquito host defence signaling in *Aedes albopictus*-derived U4.4 cells during the acute phase of infection. SFV did not activate but effectively inhibited STAT-, IMD- and Toll-mediated host defence signaling in mosquito cells and reduced host gene expression. This is similar to SFV infection of vertebrate cells, but the reduction in gene expression was less and the infection did not result in cell death. Stimulation of pathways involving STAT and IMD (but not Toll) responses before infection reduced viral gene expression and RNA synthesis, indicating that these pathways can activate antiviral activities.

## Results

### SFV4 readily establishes infection of U4.4 mosquito cells

**Figure 1:**
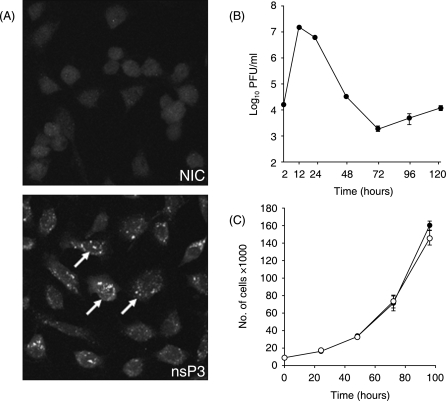
(A) Infection of U4.4 mosquito cells with SFV4. At 18 h post-infection (p.i.) cells were fixed and stained using an anti-nsP3 antibody. Replication complexes (bright focal staining) are indicated by arrows. NIC: non-infected control. (B) Growth of SFV4 on U4.4 cells. Cells were infected at a m.o.i. of 10 and virus production measured during time periods p.i., as indicated. Medium was replaced with fresh culture medium at the end of each period to measure virus production at specific time intervals. (C) Characteristics of mosquito cells infected with SFV4 (m.o.i. 10). Infected and uninfected U4.4 cell numbers were counted at regular intervals up to 96 h. 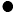: uninfected cells; 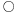: infected cells.

Early studies on SFV in mosquito cells showed that, like other arboviruses, SFV (strain unknown) infection of cultured *Ae. albopictus* cells begins with an acute phase of efficient virus production between 12–24 h, and then enters a persistent phase during which only few cells (1–2%) produce virus (Davey & Dalgarno, 1974). Persistently infected mosquito cell cultures can be maintained for many passages. For Sindbis virus, the *Ae. albopictus*-derived U4.4 cell line closely resembles infectivity in the mosquito and has been used to study virus/host interactions (Riedel & Brown, 1979; Condreay & Brown, 1986; Condreay & Brown, 1988; Miller & Brown, 1992). To study innate defence mechanisms in SFV-infected U4.4 cells, we first determined whether SFV4 (derived from the SFV prototype cDNA clone) could establish infection of these cells. Indirect immunofluorescence was used to verify SFV4 infection. At a multiplicity of infection (m.o.i.) of 10, approx. 100% of cells were infected at 18 h post-infection (p.i.) (Fig. 1A). Infection was productive; high levels of virus production were observed between 0 and 12, and 12 and 24 h p.i. (Fig. 1B). Thereafter, virus production dropped to low levels as the culture entered the persistent phase of infection. This pattern of infection was similar to that described in the earlier experiments of Davey & Dalgarno (1974) and to that described for Sindbis virus-infected U4.4 cells (Mudiganti *et al*., 2006). Cell numbers of infected and uninfected cultures increased at similar rate throughout the observation period of 96 h p.i. (Fig. 1C). Thus, SFV4 displays the expected characteristics of an arbovirus in these mosquito cells.

### SFV4 infection down-regulates host gene expression in U4.4 cells

Interference with host gene expression is one mechanism by which alphaviruses interfere with host responses in vertebrate cells (Aguilar *et al*., 2007; Breakwell *et al*., 2007; Garmashova *et al*., 2007). A previous report (measuring ^3^H-uridine incorporation into cellular RNA) showed that host RNA synthesis is inhibited 1.5- to 1.7-fold in Sindbis virus-infected *Ae. albopictus*cells (Sarver & Stollar, 1977), and led us to investigate if there is also inhibition of host gene expression in SFV-infected mosquito cells.

**Figure 2:**
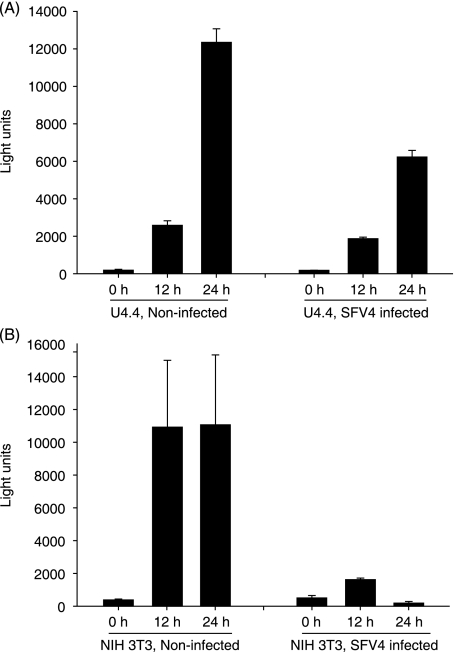
Effect of SFV4 infection on mosquito cell gene expression during the acute phase of virus production. Shut-off of host gene expression was measured using a *Renilla* luciferase reporter gene under control of a constitutively active RNA polymerase II-promoter (pGL4.75) in mosquito (U4.4) (A) and mammalian (NIH 3T3) cells (B); luciferase activities were determined at 0, 12 and 24 h p.i. Each bar represents the mean of three independent biological replicates; error bars indicate the standard deviation. Every experiment was repeated at least twice under the same conditions.

Reporter genes expressed from constitutively active promoters have been shown to reflect host gene expression in arbovirus-infected vertebrate and mosquito cells (Kohl *et al*., 2004; Leonard *et al*., 2006). To analyse host gene expression, U4.4 cells were transfected with a RNA polymerase II-dependent reporter plasmid, pGL4.75 (*Renilla* luciferase expression under the control of a CMV promoter), infected with SFV4 (m.o.i. 10) immediately post-transfection and *Renilla* expression measured during the acute phase of virus production. For comparison, *Renilla* expression was also measured in infected and uninfected mouse NIH 3T3 cells. As shown in Fig. 2A, SFV4 infection caused a moderate but consistent and significant reduction in *Renilla* activity in U4.4 cells (2-fold at 24 h p.i.), but a strong reduction in NIH 3T3 cells (7-fold at 12 h then 50-fold at 24 h p.i.) (Fig. 2B). As U4.4 and NIH 3T3 cells are infected at the same m.o.i. and all cells were infected, those differences are not due to cell numbers. Interestingly, reporter gene expression was reduced by the same magnitude at 48 h p.i., suggesting that biologically active virus proteins are still present as the culture becomes persistently infected.

### SFV4 infection of U4.4 mosquito cells does not activate the STAT, IMD or Toll signaling pathways and activation of these pathways is strongly reduced in virus-infected cells

**Figure 3:**
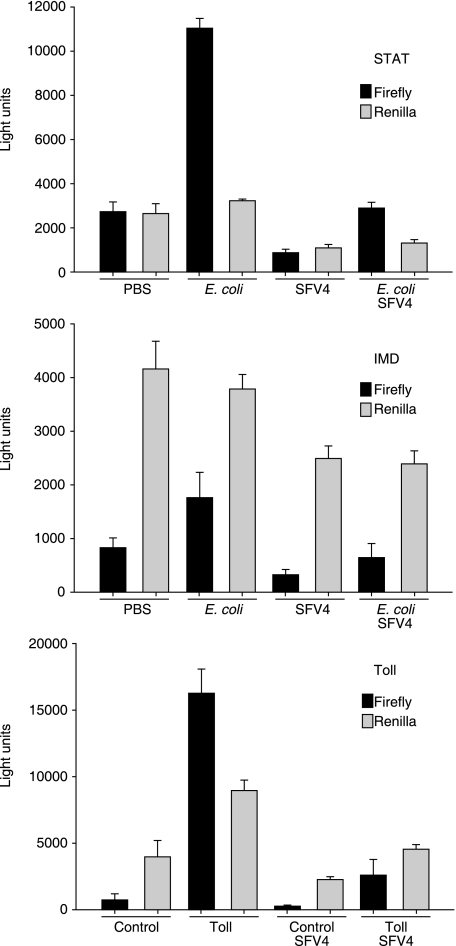
Stimulation and effect of SFV4 infection on mosquito cell defence signaling. U4.4 cells were co-transfected with pAct-*Renilla* (*Renilla* expressed under control of the RNA polymerase II-dependent *Drosophila* actin 5C promoter) as internal control and plasmids containing the STAT (p6x2DRAF-Luc)- or IMD (pJL169)- or Toll (pJM648)-responsive promoters directing Firefly luciferase expression, then immediately infected with SFV4 (m.o.i. 10) or mock-infected. Where indicated, host response pathways were then stimulated by adding heat-inactivated *E. coli* (STAT, IMD stimulation; mock: PBS) immediately post-transfection, or immediately post-infection. The Toll pathway was stimulated by transfection (simultaneously with reporter genes for Toll pathway activation) of constitutively active receptor Toll ΔLRR in pJL195 (‘Toll’) or empty insect cell expression vector pIB-V5/His (‘control’), followed immediately by infection with SFV4 (m.o.i. 10) or mock-infection. Cells were lysed and luciferase activities determined at 18 h p.i. Each bar represents the mean of three independent biological replicates; error bars indicate the standard deviation. Every experiment was repeated at least twice under the same conditions.

In arthropods, the STAT, IMD and Toll signaling pathways are known to be activated by bacteria and fungi. It is not known whether these pathways are activated by arboviruses. Dual luciferase reporter assays were used to determine the ability of SFV4 to activate these pathways. U4.4 cells were co-transfected with constitutively active internal reporter plasmid pAct-*Renilla* (expressing *Renilla* luciferase) and one of three plasmids encoding Firefly luciferase under the control of an inducible, pathway-responsive promoter. The Firefly luciferase-expression plasmids p6x2DRAF-Luc, pJL169 and pJM648 containing respectively promoters for STAT-, IMD- or Toll- inducible signaling pathways were used. Each of these dual luciferase reporter assays used a different Firefly plasmid generating different background levels of luciferase expression and different ratios of Firefly to *Renilla* luciferase expression (Fig. 3). For cells transfected with the STAT or IMD signaling pathway reporters, addition of heat-inactivated *Escherichia coli* activated both pathways (relative to PBS controls). In contrast, SFV4 infection did not activate either pathway and, consistent with the previously observed reduction in host gene expression (Fig. 2), background levels of both reporter genes were reduced. Addition of heat-inactivated bacteria to the virus-infected cells activated both the STAT and IMD pathways, demonstrating that these virus infected cells were still capable of responding to other pathogenic stimuli (Fig. 3); the magnitude of this response was however far less than that of uninfected cells. To activate the Toll pathway, which cannot be activated by *E. coli* or *Staphylococcus aureus* in U4.4 mosquito cell culture (our observations), an expression plasmid (pJL195) for a constitutively active Toll receptor (Toll ΔLRR) (Tauszig *et al*., 2000) was co-transfected with the luciferase reporter plasmids. This strongly activated the Toll pathway, > 15-fold (Fig. 3), whereas empty insect cell expression vector (pIB-V5/His) did not. Again, SFV4 infection did not activate this pathway but reduced the background level of reporter genes. SFV4 infection of Toll ΔLRR-expressing cells dramatically reduced activation of the Toll signaling pathway. Internal *Renilla* controls were consistently expressed at slightly higher levels when the Toll pathway was activated; the reason for this is not known. Taken together, these results show that SFV4 infection of U4.4 cells does not activate the STAT, IMD or Toll pathways and that infection strongly reduces the level of signaling induced by these pathways; this is possibly due to down-regulation of host cell gene expression.

### Activation of host defence signaling pathways prior to virus infection suppresses viral gene expression

**Figure 4:**
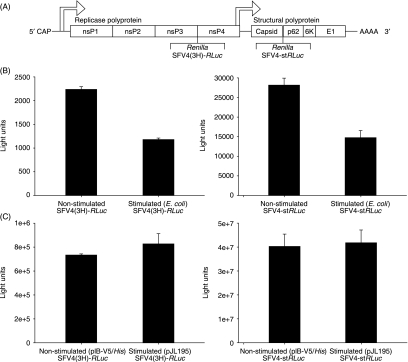
Effects of host cell defence signaling on SFV gene expression. (A) Recombinant SFV4-derived viruses expressing *Renilla*luciferase (*Rluc*) from the non-structural (SFV4(3H)-*Rluc*) or structural region of the genome (SFV4-st*Rluc*). (B) U4.4 cells were stimulated with heat-inactivated *Escherichia coli* for 1 h or mock-treated, then infected (m.o.i. 1) with recombinant SFVs. Luciferase expression was measured at 12 h p.i. (C) U4.4 cells were transfected with constitutively active Toll ΔLRR receptor (pJL195) or mock-stimulated (transfection of pIB-V5/His; empty insect cell expression vector) for 24 h then infected with (m.o.i. 1) SFV4(3H)-*Rluc* or SFV4-st*Rluc*. Cells were lysed 12 h p.i. and luciferase activities determined. Each bar represents the mean of three independent biological replicates; error bars indicate the standard deviation. Every experiment was repeated at least twice under the same conditions.

That SFV4 infection of U4.4 cells does not activate the STAT, IMD or Toll signalling pathways could result from lack of triggering or effective suppression. Whether responses initiated by these signaling pathways are able to affect arbovirus infection is not known. To allow quantitative assessment of early and late virus gene expression, recombinant viruses expressing *Renilla* luciferase from either the replicase or the structural open-reading frame of SFV4 were constructed (Kiiver *et al*., 2008). *Renilla* luciferase expressed from the genomic promoter was flanked by nsP2-protease cleavage sites at the C-terminus of nsP3 in SFV4(3H)-*Rluc*; *Renilla* luciferase expressed from the subgenomic promoter was inserted between the capsid and the envelope glycoproteins and released from the latter by inclusion of a self-processing foot and mouth disease virus 2A peptide (SFV4-St*Rluc*) (de Felipe *et al*., 2006) (Fig. 4A).

To test the effects of immune signaling involving STAT and IMD on SFV gene expression, U4.4 cells were pre-incubated for 1 h with heat-inactivated *E. coli* or with PBS, then infected with SFV4(3H)-*Rluc* or SFV4-St*Rluc*. As shown in Fig. 4B, bacterial activation of host defences prior to infection reduced virus gene expression both from the genomic and the sub-genomic promoters by around 50%. Immunostaining of parallel cultures demonstrated that the percentage of virus-infected cells in both the bacterial-treated and PBS control-treated cells were identical (data not shown), indicating that prior stimulation with bacteria did not affect virus entry. Expression of constitutively active Toll receptor (Toll ΔLRR) for 24 h, followed by infection with *Renilla*-expressing SFVs had no effect on virus gene expression at 12 h p.i. (Fig. 4C). Activation of Toll signaling before IMD/STAT signaling did not reverse the inhibitory effects of bacterial stimulation on viral gene expression (not shown).

### Stimulation of host defences involving STAT/IMD reduces virus RNA replication rates

**Figure 5:**
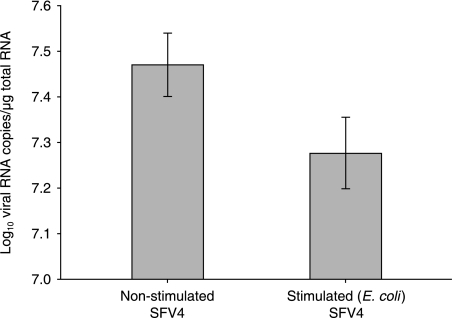
Activation of host cell defence signaling inhibits SFV4 RNA replication. U4.4 cells were treated with heat-inactivated *Escherichia coli* for 1 h (to stimulate signaling pathways involving STAT/IMD) or mock-treated, then infected with SFV4 (m.o.i. 1) for 12 h before RNA isolation. Viral genome copy numbers, which also act as viral polyprotein mRNA and are thus linked to viral gene expression, were measured by real time quantitative PCR targeting a region of nsP3. RNAs from three independent biological replicates (for both non-stimulated and stimulated cells) were reverse transcribed in triplicate; each reverse transcription reaction was then amplified in triplicate. Each bar represents the average of all measurements from a representative real time quantitative PCR experiment (see Experiemental procedures); error bars represent the standard deviation. Every experiment was repeated at least twice under the same conditions.

To determine whether viral RNA replication was affected by prior simulation with heat-inactivated bacteria (activating pathways involving STAT/IMD signaling), viral genome copies (which also serve as mRNA) were measured by real time quantitative PCR. As previously, U4.4 cells were stimulated with heat-inactivated *E. coli* for 1 h, followed by infection with SFV4 for 12 h, and total RNA was isolated. As shown in Fig. 5, bacterial stimulation reduced viral genome copy number by 40–60% (compared to non-stimulated cells), similar to the reduction in viral gene expression, demonstrating that bacterial activation of cellular defences interferes with virus RNA replication. The magnitude of this effect is similar to the effect of type I interferon on SFV RNA synthesis in vertebrate cells (Mecs *et al*., 1967).

## Discussion

Relatively little is known about arbovirus interactions with mosquito host defences. In vertebrate cells, alphavirus infections result in a reduction of host cell gene transcription which includes at least some host defence responses (Garmashova *et al*., 2006; Aguilar *et al*., 2007; Breakwell *et al*., 2007; Garmashova *et al*., 2007). Sindbis virus infection of an *Aedes albopictus* cell line different to that used in the present study reduced host RNA levels 1.5 to 1.7-fold (Sarver & Stollar, 1977). This suggests that transcriptional inhibition can also take place in alphavirus-infected mosquito cells. Our results show that alphavirus inhibition of mosquito cell gene expression occurs in SFV4-infected U4.4 cells, and is similar in magnitude to the previous observation.

Direct comparison of cellular gene expression in U4.4 and NIH 3T3 cells infected with SFV4 demonstrated that reduction of cellular gene expression was far greater in mouse (50-fold at 24 h p.i.) than mosquito cells (2-fold). Our studies extend previous work by demonstrating that SFV4-infected mosquito cells make only limited responses to stimulation of the STAT and IMD pathways with heat-inactivated bacteria, and Toll pathway-induced gene expression is also strongly reduced by SFV4 infection.

In U4.4 cells, there was no activation of the STAT, IMD or Toll pathways by SFV4 infection. Possibly this is because virus infection does not trigger these pathways. Alternatively, virus might trigger these pathways but no gene expression results due to down-regulation of host cell gene expression in virus-infected cells. Given that this down-regulation of gene expression was in the order of 2-fold, far less than that observed in vertebrate cells, and there was no indication of any upregulation of STAT, IMD and Toll pathways in virus-infected cells, it seems most likely that SFV infection does not trigger these pathways. However, we cannot rule out the possibility that SFV4 directly targets and inhibits these host defence pathways. Viruses can directly target cell defence pathways, many examples of this exist in virus/vertebrate cell interactions (Randall & Goodbourn, 2008); in mosquito cells, STAT phosphorylation is inhibited in Japanese encephalitis arbovirus (Flaviviridae) infection (similar to mammalian cells) (Lin *et al*., 2004). Venezuelan encephalitis alphavirus capsid protein does not target nuclear import in mosquito cells, while it does in vertebrate cells to interfere with host gene expression; however the experiments described would not allow to detect subtle changes in gene expression, mode of action by capsid might differ, and/or different viral proteins might be involved (Atasheva *et al*., 2008). Interestingly though, if host defence pathways, including STAT and IMD but not Toll, were stimulated prior to SFV4 infection, reporter gene expression from both the virus replicase and structural open-reading frames was reduced (50%). Virus RNA levels were also reduced (40–60%) in these cells providing an explanation, though the possibility that reduction in virus RNA levels was compounded by reductions in translation of these virus RNAs cannot be ruled out. In summary, although SFV4 infection probably does not trigger the STAT, IMD or Toll pathways, activation of host defence pathways which include STAT and IMD, but not the Toll pathway alone, does inhibit virus replication. It is possible that STAT and/or IMD pathways are the main mediators of antiviral effects against SFV4 in mosquito cells, though other pathways responsive to Gram-negative bacteria might also be involved. Other antiviral pathways might exist in mosquitoes, and this possibility needs to be further investigated. The pathogenic *Microplitis demolitor* bracovirus can not just inhibit Toll and IMD signaling, but also inhibits antimicrobial melanization reactions (Thoetkiattikul *et al*., 2005; Beck & Strand, 2007; Lu *et al*., 2008). Despite the evolutionary distance between *Aedes* and *Drosophila*, our results show that tools to study *Drosophila* microbe/cell interactions can also be successfully used in *Aedes*; this suggests some conservation of important physiological processes and might allow mosquito researchers to take further advantage of related invertebrate organisms.

It remains to be seen how this study compares to genomic studies suggesting (sometimes delayed) upregulation of host defence pathways in response to arbovirus infection in entire insects or specific target tissues (Medeiros *et al*., 2004; Sanders *et al*., 2005). IMD pathway activation in Sindbis virus-infected *Aedes aegypti* midguts is intriguing and might indeed have antiviral potential during natural infection (Sanders *et al*., 2005); however it remains to be seen what exactly activates this pathway. Direct analysis of infected cells, as in this study, suggests that host defence signaling is actively downregulated, but if activated prior to infection then virus gene expression and replication are reduced. Activation of these pathways in tissues might be a secondary reaction to dsRNA, cytokines, or other ‘danger signals’, as suggested for *Drosophila* X virus (Zambon *et al*., 2005). Tissue damage should not be excluded as alphaviruses can induce pathological changes in mosquitoes (Mims *et al*., 1966; Weaver *et al*., 1988; Scott & Lorenz, 1998; Bowers *et al*., 2003).

It is not yet clear how virus gene expression and replication can be affected by mosquito innate immunity, and how important these pathways are compared to the relatively slow RNAi responses. Mammalian defensins have antiviral activity at various levels (Klotman & Chang, 2006) and antiviral activities of mosquito antimicrobial peptides is possible.

In summary, host defence signaling, including the STAT, IMD and Toll pathways, is strongly reduced in SFV4-infected U4.4 cells, probably as a result of a general inhibition of host cell gene expression. These pathways are not activated in SFV4-infected cells, possibly because of this prior inhibition of host cell gene expression or alternatively because they are not triggered by this infection. Activation of pathways involving STAT/IMD but not Toll signaling prior to infection does nevertheless exert an anti-viral effect.

## Experimental procedures

### Cells and viruses

The *Ae. albopictus*-derived U4.4 cell line (Condreay & Brown, 1986) was grown at 28 °C in L-15/10% fetal calf serum (FCS)/8% tryptose phosphate broth (TPB). Strain SFV4 and recombinant viruses were grown in BHK-21 cells (37 °C; in GMEM/2% newborn calf serum (NBCS). NIH 3T3 mouse cells were grown at 37 °C in DMEM/10%NBCS/5 mM L-glutamine.

Virus-containing supernatants were clarified by centrifugation (3 ×, 30 min, 15 000 rpm) and viruses concentrated from supernatant on a 20% (w/v) sucrose/TNE buffer (pH 7.4) cushion by ultracentrifugation (25 000 rpm, 90 min, SW28 rotor). Pellets were resuspended in TNE buffer, and viruses titrated by plaque assay. Infection of mosquito and mammalian cells was performed at 28 °C or 37 °C for 1 h, respectively, at a m.o.i. of 10 plaque forming units (PFU) per cell (unless otherwise stated) in PBS/0.75% BSA. After infection complete medium was added to the cells. To establish growth curves, approx. 6.5 × 10^5^ U4.4 cells/well (in 6-well plates) were infected at a m.o.i. of 10; medium to be titrated was taken off and replaced with fresh medium for intervals indicated. For growth comparisons of infected and noninfected cells, approx. 1.3 × 10^5^ U4.4 cells/well (in 24-well plates) were infected at a m.o.i. of 10 and counted at times indicated.

### Immunostaining

U4.4 cells were fixed with 4% paraformaldehyde. After 2 washes (always in PBS), cells were permeabilised with 0.3% Triton-X100 in PBS for 20 min and washed 2x. After blocking with CAS block (Invitrogen, Paisley, UK) (20 min), primary anti-nsP3 antibody (in CAS block; anti-nsP3 1:800) was added, followed by 3 washes. Incubation with secondary antibody (goat-anti-rabbit biotinylated IgG in CAS block; 1:750) was followed by 3 washes, and streptavidin-conjugated Alexa Fluor 594 was added. After 2 washes, slides were mounted with mounting medium (Vector Laboratories, Peterborough, UK) and images acquired (Zeiss AxioSkop confocal microscope; Carl Zeiss Ltd., Welwyn Garden City, UK).

### Plasmids

Plasmid pGL4.75 (Promega, Southhampton, UK) contains the *Renilla* luciferase gene under control of the CMV IE promoter. Insect cell expression plasmid pIB-V5/His was obtained from Invitrogen. pAct-*Renilla* (Karsten *et al*., 2006) contains the *Renilla*luciferase gene under control of the *Drosophila* actin 5C promoter for constitutive expression. p6x2DRAF-Luc (Hombria *et al*., 2005) is a multimerised *Drosophila* STAT-responsive element with a Firefly luciferase reporter. IMD-pathway reporter pJL169 (Firefly luciferase under control of the *Drosophila Attacin A* promoter), Toll-pathway pathway reporter pJM648 (Firefly luciferase under control of the *Drosophila Drosomycin*promoter) and plasmid expressing constitutively active Toll receptor (pJL195; Toll ΔLRR) have been previously described (Tauszig *et al*., 2000).

### Transfection of DNA, Toll pathway activation and luciferase assays

U4.4 (650 000 cells/well, or as indicated) or NIH 3T3 (10^6^ cells/well) cells in 6-well plates were transfected using 1 µl Lipofectamine 2000 (Invitrogen) per well. Before transfection, medium in wells was replaced by fresh complete medium. Nucleic Acid/Lipofectamine 2000 complexes were prepared in OPTIMEM (Invitrogen). Amounts of DNA used: 10 ng pGL4.75 or combinations of pAct-*Renilla* (25 ng) with p6x2DRAF or pJL169 (100 ng). For Toll pathway activation pAct*-Renilla* (25 ng), pJM648 (1 µg) and pJL195 (or pIB-V5/His as negative control; 800 ng) were co-transfected (using 3 µl Lipofectamine 2000 per well) for 3 h, then fresh medium was added. Cells were lysed in Passive Lysis Buffer. Luciferase activities were measured using a Dual Luciferase assay kit (Promega) on a GloMax 20/20 Luminometer.

### Pathway stimulation with *Escherichia coli*

To prepare working stocks of *E. coli* strain JM109 (New England Biolabs, Hitchim, UK), 1 µl of bacterial stock solution was added to 5 ml LB medium (without antibiotics), incubated at 37 °C/18 h, and centrifuged (4 °C, 2500 rpm/10 min). Cells were resuspended in 0.5 ml PBS, and bacteria inactivated by heating the suspension at 80 °C for 10 min. Fresh stocks were prepared for each experiment (concentration approx. 9 × 10^5^ bacteria/µl). For transfection/stimulation experiments, 5 µl of the suspension (approx. 4.5 × 10^6^ cells) were added into 2 ml complete medium/well, post transfection (and, where indicated, infection). Assuming that volume of *Aedes* hemolymph is approx. 50 nl (Shapiro *et al*., 1986), the concentration of approx. 2250 inactivated bacteria per µl culture medium is below lethal concentrations in live *Drosophila* (Pham *et al*., 2007). To measure effects on viral gene expression, U4.4 cells (650 000 cells/well in 6-well plates) were stimulated (1 h) with heat-inactivated bacteria or mock stimulated as described above, followed by infection with recombinant SFV for 1 h. Reporter gene expression was analysed by luciferase assays at 12 h p.i.

### Real time quantitative PCR (qPCR)

Quantification of viral genome copy numbers was carried out essentially as described (Breakwell *et al*., 2007). Briefly, cultures of U4.4 cells (650 000 cells/well) were stimulated with heat-inactivated *E. coli* or mock-stimulated (3 independent biological replicates each) for 1 h as described above, followed by infection with SFV4 (m.o.i. 1) for 12 h. Following RNA extraction, quantity and quality were assessed on a nanodrop spectrophotometer (Fisher Scientific, Loughborough, UK). 0.5 µg of total RNA from each replicate were reverse-transcribed (again in triplicate), and each of those reactions was analysed in triplicate by qPCR. The reaction mix contained the following: 0.8 µM of each primer, 40 mM deoxynucleoside triphosphates, 3 mM MgCl_2_, 1:10 000 SYBR Green (Biogene Ltd., Kimbolton, UK), 0.75 U Fast Start *Taq* (Roche Applied Science, Burgess Hill, UK), and 2 µl of template. Tubes were heated to 94 °C for 5 min, and the PCR was then cycled through 94 °C for 20 s, 62 °C for 20 s, and 72 °C for 20 s for 40 cycles on a RotorGene 3000 instrument (Corbett Research, St. Neots, UK). Sequences of the primers were as follows: SFV-nsP3-for 5′-GCAAGAGGCAAACGAACAGA-3′, SFV-nsP3-rev 5′-GGGAAAAGATGAGCAAACCA-3′.
